# High-fidelity spherical cholesteric liquid crystal Bragg reflectors generating unclonable patterns for secure authentication

**DOI:** 10.1038/srep26840

**Published:** 2016-05-27

**Authors:** Yong Geng, JungHyun Noh, Irena Drevensek-Olenik, Romano Rupp, Gabriele Lenzini, Jan P. F. Lagerwall

**Affiliations:** 1 University of Luxembourg, Physics & Materials Science Research Unit, L-1511 Luxembourg, Luxembourg; 2 University of Ljubljana, Faculty of Mathematics and Physics, Jadranska 19, SI 1000 Ljubljana, Slovenia; 3 J. Stefan Institute, Jamova 39, SI 1000 Ljubljana, Slovenia; 4 Faculty of Physics, University of Vienna, A-1090 Vienna, Austria; 5 University of Luxembourg, Interdisciplinary Center for Security and Trust, L-2721 Luxembourg, Luxembourg

## Abstract

Monodisperse cholesteric liquid crystal microspheres exhibit spherically symmetric Bragg reflection, generating, via photonic cross communication, dynamically tuneable multi-coloured patterns. These patterns, uniquely defined by the particular sphere arrangement, could render cholesteric microspheres very useful in countless security applications, as tags to identify and authenticate their carriers, mainly physical objects or persons. However, the optical quality of the cholesteric droplets studied so far is unsatisfactory, especially after polymerisation, a step required for obtaining durable samples that can be used for object identification. We show that a transition from droplets to shells solves all key problems, giving rise to sharp patterns and excellent optical quality even after polymerisation, the polymerised shells sustaining considerable mechanical deformation. Moreover, we demonstrate that, counter to prior expectation, cross communication takes place even between non-identical shells. This opens additional communication channels that add significantly to the complexity and unique character of the generated patterns.

Ever since their discovery, cholesteric liquid crystals have fascinated their observers thanks to their striking iridescent and dynamic colours^1^, changing sensitively in response to external influences like temperature variations, mechanical pressure, shear flow, or electromagnetic fields. The colours are due to optical Bragg reflection from a spontaneously formed helical structure with a pitch *P* on the order of visible light wavelengths, which turns cholesterics into dynamically tuneable, self-assembled photonic crystals^2^. Within a band gap Δ*λ* = Δ*nP* around the central reflection wavelength 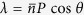, circularly polarised light with the same handedness as the cholesteric helix cannot propagate but is fully reflected. Here Δ*n* is the birefringence, 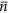 is the average refractive index and *θ* is the angle between the light propagation direction and the helix axis.

With recent advances in microfluidic technology it has become possible to prepare large quantities of monodisperse droplets or shells of cholesterics. These unusual microspheres display unique as well as highly useful optical properties thanks to the combination of spherical symmetry and optical Bragg reflection^3^,^4^,^5^,^6^,^7^,^8^,^9^,^10^,^11^,^12^,^13^. Humar and Musevic succeeded in turning cholesteric droplets into omnidirectional lasers^3^ and later Uchida *et al*.^4^ and Chen *et al*.^5^ achieved the same with cholesteric shells. Cipparrone and co-workers demonstrated chiral optomechanics involving the interaction of cholesteric droplets and optical tweezers^6^,^7^, and we recently demonstrated and explained how a new type of photonic cross communication develops between monodisperse cholesteric droplets, giving rise to intriguing multi-coloured patterns that are highly tunable^8^. Several groups have demonstrated interesting variations of the latter theme, including photo-tuning of the colour^9^ and/or the polarisation^10^ of the reflected light, as well as encapsulation of cholesteric droplets in hydrogel or rubber shells^11^,^12^.

We foresee several useful applications of these remarkable systems^8^. For instance they can be used as photonic couplers, all-optical switches/distributors, or autonomous sensors. Of particular current interest is their potential use in security. Indeed, a collection of cholesteric liquid crystal spheres seems to be a natural candidate for realising—due to its unique optical characteristics, the practical impossibility to copy a certain arrangement of such microspheres, and the fact that any attempt at tampering with the arrangement will likely ruin the structure—what in cryptography is known as a Physical Unclonable Function (PUF)^14^,^15^. This is a physical object that, because of an intrinsic randomness present within its manufacturing process, inherently possesses unique, unpredictable and unclonable challenge–response combinations. Such a quality can be used to uniquely identify an object, assuming that the response can be extracted from the object with high fidelity and represented digitally^16^.

The inherent randomness of a cholesteric microsphere-based PUF would come primarily from a stochastic arrangement of shells of slightly different types, as described in detail below. Other random factors, such as fluctuations in shell diameter and thickness, can additionally be introduced to increase the inherent entropy and to render each arrangement unclonable and capable of generating a unique and, we hypothesise, unpredictable optical pattern. A PUF based on cholesteric liquid crystal shells would have a wide spectrum of practical uses in security. Incorporated in a plastic film appropriately attached to an object of high value, it can be used to identify its carrier, thus finding application in the design of anti-counterfeiting mechanisms; it can be used to trace valuable goods (e.g., artwork, jewellery, documents) and dangerous or sensitive items (e.g., toxic waste, medicaments) and to prove their authenticity, because—as we describe in this paper—the shells can be made such that they would break in case of an attempt to transfer the PUF to a copy, thereby carrying evidence of the tampering. Applied as a temporary tattoo or printed onto clothes, a PUF of this design could be worn by people (e.g. soldiers and volunteers working in life-threatening places) and used as a proof of identity.

Because they react to luminous stimuli with an optical pattern that is assumedly unpredictable (random) at the production stage, cholesteric liquid crystal spheres could also become a security component in object-entangled cryptography devices and used to generate encryption keys for remote authentication over the Internet. Their detailed security characteristics need to be assessed in future work and so do aspects like robustness/stability in reproducing patterns when exposed to identical stimuli. However, once such a study is accomplished and these features established, the application of cholesteric liquid crystal microspheres in secure identification and authentication seems—considering that they can be produced easily, quickly and without prohibitive cost—of enormous potential.

Before we can realistically exploit these novel photonic materials in security, however, the optical quality of each cholesteric sphere needs to be improved and the liquid crystal needs to be transformed into a material that is appropriately durable and easily processable, e.g. via polymerisation^17^,^18^. Aβhoff *et al*.^10^ explored the cross communication between cholesteric droplets after polymer-stabilisation (other work studying polymerized cholesteric droplets^6^,^7^ only investigated single-droplet properties), finding that the optical quality was severely degraded by textural defects induced by the polymerisation. This is understandable, considering that the volume to be polymerised in such a droplet is substantial, with a diameter two orders of magnitude larger than the characteristic distance of typical liquid crystal devices, and since alignment is provided only by the external surface. Although the overall cross communication pattern was detectable, the authors concluded that a different strategy for making cholesteric microspheres durable was needed.

In this paper we explore the full range from droplets to shells of cholesteric liquid crystals, finding that the optical quality, as well as the possibility to polymerise the sphere with retained photonic performance, is greatly enhanced by moving to thin shells rather than droplets. Applying an osmotic thinning procedure to the shells, we also significantly bring down the annealing time required to expel commonly generated defects that are detrimental for the optical properties. Finally, we extend the analysis of the photonic cross communication, taking into account the spread in reflection directions and wavelengths that applies to these systems. This allows us to further advance the uniqueness and complexity in the generated patterns by mixing shells of cholesterics with different pitch. We demonstrate that this does not lead to terminated photonic cross communication, as the original analysis would suggest, but instead to a new communication mode at intermediate wavelengths, enhancing substantially the attractiveness of the system for security applications.

## Results

### Preparation and characterisation of fully fluid cholesteric liquid crystal shells

**Figure 1:**
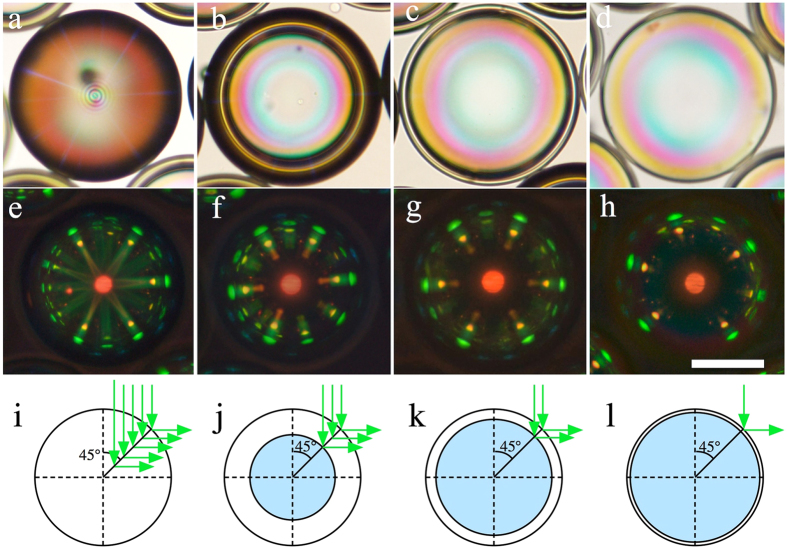
Photonic cross communication patterns as a function of shell thickness. The top row shows each droplet/shell in transmission without analyser (**a**–**d**), while photos (**e**–**h**) show the corresponding reflection images between crossed polarisers. In **a**/**e** the sphere is a droplet, in the other cases it is a shell, with thickness 40 μm (**b**,**f**), 15 μm (**c**,**g**) and 5 μm (**d**,**h**), respectively. The scale bar is 100 μm. Schematic drawings at the bottom illustrate to what extent internal reflections occur at a cholesteric helix inclined at 45° to the incidence direction, explaining why the pattern becomes less diffuse as the thickness decreases.

Figure 1 shows the optical appearance of well-aligned cholesteric spheres with radii about 100 μm, each surrounded by identical spheres arranged more or less in a close-packed hexagonal pattern, for the cases of droplets (left column) and shells of thickness decreasing stepwise from left to right, from 40 μm to 5 μm. In all four cases the hexagonal symmetry of the overall sphere arrangement is clearly seen in the reflection pattern, because of the photonic cross communication taking place between the spheres^8^. When light incident from above is Bragg-reflected by a nearby sphere into the horizontal plane, the pictured sphere reflects this light back up to the observer. For vertically incident light this means that the reflection angle *θ* must be 45°, i.e. the light wavelength must fulfill 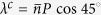, where we let the superscript c denote direct cross communication. For the liquid crystal mixture in Fig. 1, 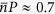 μm, corresponding to the red spot at the centre of the sphere, arising from normal incidence reflection. This means that the horizontally reflected light of direct cross communication has a wavelength of about 0.5 μm, corresponding to the green spots seen along the perimeter. A second category of cross communication gives rise to yellow spots, with the same hexagonal symmetry as the main green spots, a bit further towards the centre of each sphere. This communication is indirect, mediated via a total internal reflection (TIR) event at the top surface of the continuous phase^8^, which in these uncovered samples is followed by air. Several additional series of spots can arise from cross communication with cholesteric spheres situated further away from the central one, in particular in uncovered samples with rather large spheres^8^,^19^.

It is clear that the thinnest shell offers by far the sharpest reflection pattern. The patterns from thicker shells and from the droplet are blurred by unfocused reflections, originating in light entering at locations where the helix is not in reflection orientation at the surface, as explained by Fan *et al*.^9^. This light is then admitted into the interior until it reaches a point where the helix axis has the 45° angle orientation required for horizontal reflection, cf. the bottom row of Fig. 1. Because of the simultaneous lateral and vertical shifts of the reflection point, the resulting picture shows diffuse radial lines rather than the desired sharp spots that characterise the thin shell in Fig. 1h.

In order to reach complete Bragg reflection of the wavelength and polarisation that match the cholesteric helix, a sample thickness of about ten helix pitches is required^20^, or about 5 μm. This condition is met for the thinnest shell in Fig. 1, hence there is no benefit of making the shell thicker.

### Defect removal by osmotic thinning

There is, however, a significant gain in producing the shell with somewhat greater initial thickness, and then reducing it by osmotic thinning. Because the liquid crystal is not impenetrable to water^21^ the shell acts as a semipermeable membrane across which an osmotic pressure develops if the inner and outer aqueous phases have different compositions. This pressure can be used to expand and thin the shell in a controlled manner^22^, with great benefit for the optical properties. Immediately after production, droplets as well as shells are of poor optical quality, because the cholesteric is then rich in so-called oily streak defects^23^. These are of symmetric type, which are unstable and disappear over time^24^, but this is a slow process. In order to obtain the photos in Fig. 1, the samples had to be annealed at room temperature for about seven days.

**Figure 2:**
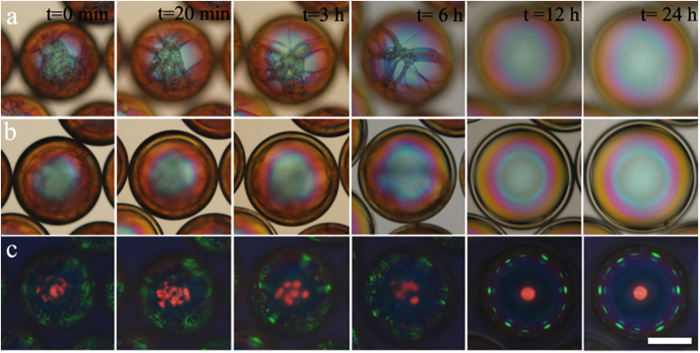
Removal of oily streak defects by osmotic thinning. Optical microscopy textures in transmission without analyser (**a,b**; focus on top and centre plane of shell, respectively) and in reflection between crossed polarisers (**c**) of a cholesteric shell subjected to expansion and thinning by osmosis. The scale bar is 100 μm. After some 12 hours of osmotic expansion the shell is free of visible defects, as compared to the week-long annealing required without osmosis.

In an improved approach, we transferred shells prepared with an inner water-glycerol solution of polyvinyl alcohol (PVA) into a new outer aqueous phase with substantially lower PVA and glycerol content, giving rise to an osmotic flow of water into the internal aqueous droplet. As a result, the shells expanded and got thinner over the course of several hours, with a consequent rapid disappearance of oily streak defects (Fig. 2). Already after 12 hours the shells are free of visible defects, with an excellent cross communication pattern developing when the sample is viewed in reflection (Fig. 2, row c). The inner set of yellow TIR cross communication spots seen in Fig. 1 is absent here, a result of the change from air to glass for the phase external to the continuous phase^10^. In the process of osmotic expansion, the shell diameter increased from about 180 μm to about 240 μm, and the shell thickness decreased from 27 μm to 11 μm.

### Polymerisation of cholesteric liquid crystal shells

Another very important advantage of using shells rather than droplets is that the achieved uniform alignment is more robust, thanks to the tight confinement of a small volume of cholesteric between two closely spaced tangential-aligning interfaces. A cholesteric shell can thus be (partially) polymerised without any noticeable degradation of order, with fully retained optical quality. This is in stark contrast to the case of droplets, which suffer from severe defect generation during polymerisation, even at only 4.7% reactive component^10^. To turn the shell completely into a solid (which may actually not be desirable, as discussed below), all components of the cholesteric mixture would need to be replaced by reactive molecules^25^. However, even using just a single achiral reactive mesogen we can polymerise a sufficient fraction of the shell to ensure robustness against mechanical shock and shells pushing against each other, thereby allowing easy manipulation and greatly expanding the application opportunities for the shells. Without any polymerised component, shells easily break upon manipulation, and upon too close contact, adjacent shells collapse and merge into a single droplet.

**Figure 3:**
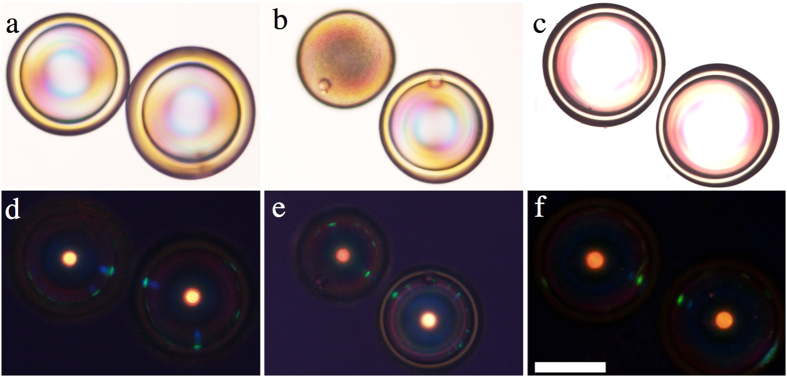
Polymer-stabilised shells with retained high-fidelity photonic cross communication. All samples contain 5% RM-257 by weight. The spheres in the first two columns were polymerised rapidly (100% UV light intensity for 1 min.) whereas the right-most sample was polymerised slowly (10% for 10 min.). The upper left-hand sphere in (**b,e**) is a droplet, all others are shells. Samples were filled into a glass capillary for investigation (thus no TIR communication). The top row shows transmission textures without analyser, the bottom row the reflection texture between crossed polarisers. The scale bar is 100 μm.

We used the commonly employed commercially available reactive mesogen RM-257, added at a fraction between 5 and 20 wt.-% to our basic cholesteric mixture, as well as a photoinitiator to start the polymerisation by UV light irradiation. The sample contained a few droplets in addition to the shells, allowing us to compare the response to polymerisation in shells and droplets. Three examples of polymerised samples (prepared with 5% RM-257) are shown in Fig. 3, in transmission without analyser in the top row and in reflection between crossed polarisers in the bottom row. The sample in the second column contains a droplet next to a shell. The first two cases (a-b and c-d) were polymerised rapidly (1 min. at 100% UV intensity) while the process in the third sample (c/f) was slow (10 minutes at 10% UV intensity). In all cases, the optical quality of the shells after polymerisation is excellent, with very well defined optical communication and intense main reflection from the shell centre and no apparent defect generation seen either in transmission or reflection. A slight blue shift in the reflection wavelength can be noted, originating in the small degree of shrinkage that is always induced by polymerisation^26^. The droplet, in contrast, scatters light strongly in its final state, a result of considerable defect generation during polymerisation. In reflection, the intensity of the central main reflection is weakened compared to the shell, reflecting the reduced degree of order in the droplet.

**Figure 4:**
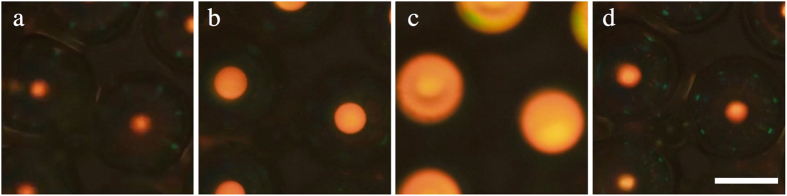
Robustness against mechanical pressure of polymer-stabilised cholesteric shells. Shells with ideal photonic properties can be produced with 20 wt.-% RM-257. After polymerisation these shells sustain strong mechanical pressure ((**a**) no deformation; (**b**) shells compressed until the top is in the focal plane; (**c**) maximum compression during the experiment), as well as shells pressing against each other. After releasing the pressure (**d**) the photonic cross communication is immediately recovered. The images are obtained in reflection between crossed polarisers and extracted from Movie SI5 in the Supporting Information; scale bar: 100 μm. The continuous distortion of the sample, with consequent variations in location of the reflection plane, renders all photos except (**b**) somewhat out of focus.

Already 5% reactive component contributes significantly to the mechanical robustness of the shells, but higher fractions obviously provide further stabilisation. The shells in Fig. 4, containing 20% RM-257, were deformed mechanically after polymerisation by pressing on a cover slip over the sample. The shells are close enough that they touch and push against each other during the process. Snapshots from the experiment are included in the figure and the whole experiment is in Movie SI5 in the Supporting Information. Although the shells are distorted so strongly that the central reflection spot increases almost 20-fold in area, the cross communication pattern is fully retained immediately after the pressure is removed, as shown in pane (d). Importantly, these shells carry no protective coating, in contrast to the system studied by Lee *et al*.^11^, simplifying sample production and also enhancing the optical properties, since any additional layer will introduce reflection and scattering.

### Photonic cross communication between non-identical shells

**Figure 5:**
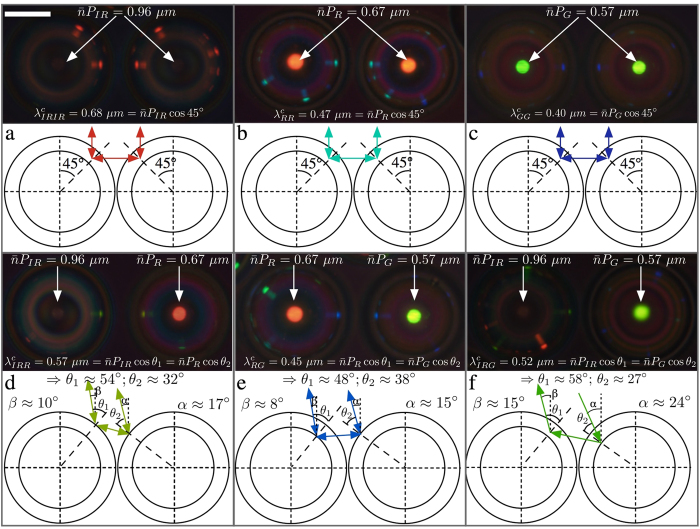
Photonic cross communication between shells with identical (**a–c**) and different (**d–f**) helix pitches. The top part of each pane shows the reflection polarising microscopy texture together with the equations governing the reflection wavelengths (determined spectrophotometrically, see Supporting Information), while the schematics underneath illustrate communication pathways. The scale bar in (**a**) is 100 μm. No TIR spots appear since the sample is studied within a glass capillary.

We end the Results section by demonstrating that very interesting new cross communication patterns can be generated by mixing shells made from cholesterics with different pitch. Based on the simplest level of analysis presented in our original work^8^, shells with different pitch should not communicate, and one would thus expect missing spots in the communication patterns between shells with different pitch. In reality, however, cross communication spots do appear, as shown in Fig. 5d–f, and their colours reflect communication at intermediate wavelengths compared to the ordinary 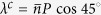 in case of identical shells (Fig. 5a–c). The three different pitch lengths investigated were such that their normal incidence reflections, seen in the central spot in each shell, are centred at the wavelengths 0.96 μm (infrared, IR), 0.67 μm (red, R) and 0.57 μm (green, G), respectively.

As illustrated in the schematics below each micrograph in Fig. 5 (the detailed calculation can be found in the Supporting Information), the communication between shells with different *P* becomes possible because the asymmetry in cholesteric pitch can be compensated by an asymmetry in ray directions and reflection planes. In our earlier work^8^, we considered only surface reflections under precisely vertical illumination, neglecting the facts that light may enter into the shell and get reflected below its surface (as discussed in connection to Fig. 1) and that there is a spread in directions of the light rays involved in generating the pattern. The microscope used for illuminating and imaging the sample integrates light over a certain range of propagation directions, within cones with opening angles γ_i_ and γ_r_ for illuminating and reflected light, respectively. The size in air of the imaging cone is given by the numeral aperture (N.A.) of the objective, via the formula *N.A.* = *n* sin *γ*_*r*_. Inside the sample this corresponds to a cone with a slightly smaller opening angle γ_r_′, due to refraction at the interface between air and the glass capillary, with refractive index *n* ≈ 1.5. All internal refraction can be neglected since also the liquid crystal and continuous phase have 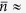 *n* ≈ 1.5. With N.A. = 0.45, yielding γ_r_ = 26.7°, and Snell’s law, 1.5 sin *γ*_*r*_′ = sin*γ*_*r*_, we obtain γ_r_′ ≈ 17.5° (see Supporting Information for details). A similar reduced cone with opening angle γ_i_′ applies for the illumination.

For clarity, only one exemplary path of cross communication is drawn for each case in Fig. 5d–f, but in reality there is a small range of permitted ray directions, wavelengths and reflection planes for each shell combination. This is derived in the Supporting Information, providing also an interactive simulation where the communication path can be tuned dynamically. As a consequence, there is a certain spatial extension of the communication spots and some wavelength variation as well. The range of admissable mismatch between the pitches of communicating shells is fundamentally limited by γ_i_′, γ_r_′ and by the thickness of the shell.

In the experiment in Fig. 5, cases (d) and (e) are within the bidirectional communication range, as the incoming and outgoing beams, within the sample, have angles α and β with respect to the vertical direction that are clearly less than γ_r_′. Curiously, the situation in Fig. 5f leads to *unidirectional* cross communication, with only one communication spot in the long-pitch shell, whereas the short-pitch shell shows no sign of communication with the neighbour. We see in the ray tracing drawing below the photo that the Bragg reflection angles θ_1_ and θ_2_ can only be realized if at least one of the angles α or β is substantially larger than γ_r_′. The numerical aperture value of the objective used is specified for the imaging light cone, whereas the value γ_i_′ of the illumination light cone can be regulated with an aperture diaphragm, being effectively even larger when the aperture is fully open. In this way we could achieve γ_r_′ < α = 24° < γ_i_′, allowing the communication to take place from right to left in the photo, but not in the opposite direction.

#### Discussion

##### Suitability of polymer-stabilised cholesteric shells for secure authentication

The photonic cross communication between shells with different values of the cholesteric pitch means that one and the same shell can exhibit many different colours of communication spots along its perimeter, as seen in Fig. 5d–f, and demonstrated more vividly in Fig. 3 in the Supporting Information. The possibility to randomly combine shells with different photonic band gaps in a single token, with bi- as well as uni-directional communication at intermediate wavelengths, thus vastly extends the possibilities of creating unique and highly distinct patterns using these shells, with great potential in the field of secure authentication. The high optical quality and sharp features of the patterns make them easier to read out with high fidelity than many other randomly generated patterns^14^, and at the same time they are practically impossible to reproduce. The reflected light is circularly polarised, and in one and the same sample left- and right-handed polarisations can be combined, as demonstrated by Aβhoff *et al*.^10^. Further security is offered by the dynamics given by the variations in pattern depending on the illuminated area^8^, and from the discussion around Fig. 5 it is clear that also a variation in illumination cone can have a dramatic effect.

With an understanding of the physics and chemistry involved and the exact same type of materials and equipment, it would be possible to produce similar shells. It might also be feasible, at least in principle, to arrange them in the same order as in an original token of the most rudimentary design, where the shells are regularly ordered in an equilibrium arrangement, given sufficient time and access to extremely precise micromanipulators. However, the latter step could easily be rendered practically impossible by solidifying the continuous phase rapidly after introducing the shells, e.g. via photopolymerisation, such that they are kinetically trapped in a non-equilibrium arrangement that could not be reproduced in a step-by-step micromanipulation process. Additional complexity could be achieved by arranging shells in multiple layers^9^ or by analysing the token in reflection as well as in transmission. The transparency and lack of scattering of the cholesteric shells gives them a characteristic appearance also in transmission, as discussed in detail elsewhere^27^.

Unclonability is an essential quality of any technology considered to realize PUFs and use them for security. We are confident that wisely prepared cholesteric shell arrangements will enjoy this feature. More precisely, we intend here physical unclonability^28^, that is, the impossibility for an adversary to produce a copy of an array of shells identical to the one s/he has entered into possession. The unclonability here derives from how the shells are allowed to arrange themselves at production within the hosting medium, with random combinations of shells with different values of the cholesteric pitch length, preferrably arrested in a non-equilibrium configuration, as just discussed, or possibly in a randomly packed arrangement^29^. Unclonability implies also that the pattern that an array of shells will produce is theoretically unpredictable at the moment of production, a criterion that our proposed random shell arrangements clearly live up to.

Unclonability can also be discussed in a non-physical sense, referring to the question of whether or not the optical pattern emerging from a specific array of shells could be reproduced via computer simulation by an adversary with limited computing resources that got access to this array. At the current stage of our research we can only hypothesize that also this feature could be met by appropriate cholesteric shell arrangements. The optical cross-communication demonstrated in this paper suggests non-local dependences in the definition of the pattern but a quantification of the degree of non-locality, thus describing how hard it is to simulate with a computer the challenge-response behavior of an array of shells, is a question that we will address in our future investigations.

An interesting aspect is that complete polymerisation of the shells may in fact not be desirable when applying them in a PUF-based authentication protocol. The ideal degree of mechanical sturdiness should be just enough to allow the shells to be processed after production, as required in order to realise the intended token with non-identical shells made from different cholesteric mixtures. After partial shell polymerisation, this could easily be done in a second microfluidic set-up, designed as to let the different shells arrange randomly, thereby generating an a priori unpredictable and unique pattern. To produce the final token, the continuous phase used at this stage would be solidified, e.g. via photopolymerisation, solvent evaporation or sol-gel processing. This token must sustain the slight compression and flexing corresponding to handling during normal use, but if it is exposed to more drastic mechanical strain the shells should in fact rupture. This will render the token tamper resistant, as an attempt to remove it from its proper enclosure will then immediately invalidate the token. In this respect, the shells with 20% polymerised component in Fig. 4, possibly even less, may be ideal. They clearly withstand the conditions of normal usage, but the abnormal deformation during an attempt to physically modify a token would lead to irreversible damage due to the partially liquid state of the shells.

Combining the ease in generating PUFs from cholesteric liquid crystal shells, that respond with dynamic, unpredictable, unique and unclonable photonic patterns to different light stimuli, with the tamper-resistance ensured by a tailored degree of polymerisation, we conclude that this new configuration of cholesteric liquid crystals constitutes an extremely interesting component for realising future high-security authentication and identification protocols. Our experiments demonstrate that the optical quality of shells is greatly enhanced compared to that of droplets, in particular after a stage of osmotic thinning of the shell, which effectively removes oily streak defects. Importantly, the optical quality is not negatively affected by the polymerisation procedure, in stark contrast to the case when droplets are polymerised. From the point of view of robustness in the optical analysis of the patterns this is important, as the defect-free state minimizes optical noise. Adding the enormous variation possibilities given by combining shells with non-identical photonic band gaps, where each combination opens a specific cross communication channel, it is clear that cholesteric micro shells have outstanding potential in security, thereby aiding to solve a problem of great current societal importance.

## Methods

### Liquid crystal materials

The cholesteric base mixtures were made by mixing the chiral dopant (S)-4-Cyano-4′-(2-methylbutyl)biphenyl (CB15, Synthon Chemicals, Germany) with the nematic mixture RO-TN 615 (Roche, Switzerland). The CB15 concentration was varied between 20 and 40 wt.-% in order to tune the cholesteric pitch as desired, the normal reflection wavelength changing from infrared to deep blue in this range. When targeting polymerisation of the shell, between 5 and 20 wt.-% of the reactive mesogen 1,4-bis-[4-(3-acryloyloxypropyloxy)benzoyloxy]-2-methylbenzene (RM-257, Merck, Korea) was dissolved in dichloromethane and added to a suitable cholesteric base mixture. The solution was heated to 75 °C under stirring for 24 hours to fully evaporate the solvent. Afterwards the photoinitiator Irgacure 2022 (Ciba, 20 wt.-% with respect to RM-257) was added to the mixture, which was shaken until uniformity.

### Shell/droplet preparation

A detailed account of the microfluidic preparation of cholesteric microspheres is provided in the Supporting Information. In brief, the liquid crystal was flowed between water-glycerol mixtures forming inner and outer phases, with polyvinyl alcohol (PVA) added as stabiliser, in a nested capillary microfluidic set-up following the basic design principles of Utada *et al*.^30^.

### Polymerisation procedure

A UVATA LED UV curing system (delivering 8800 mW/cm^2^ at full power) was used to initiate the polymerisation process. The system is equipped with an optical fibre head, which was held 2 cm from the sample, with the beam directed at 45° to the sample plane.

### Optical characterisation

A polarising microscope (Olympus BX51, Japan) equipped with a digital camera (Olympus DP73, Japan) was used for optical characterisation. The reflection wavelengths were measured by a spectrophotometer (AvaSpec-2048, Avantes) connected via fibre optics to the microscope. The size and thickness of the shells were determined from the videos/images using *ImageJ* image processing software (version 1.49V).

## Additional Information

**How to cite this article**: Geng, Y. *et al*. High-fidelity spherical cholesteric liquid crystal Bragg reflectors generating unclonable patterns for secure authentication. *Sci. Rep.* **6**, 26840; doi: 10.1038/srep26840 (2016).

## Supplementary Material

Supplementary Information

Supplementary Information

Supplementary Information
