# Supplementary material for: High-fidelity spherical cholesteric liquid crystal Bragg reflectors generating unclonable patterns for secure authentication
Source: Sci Rep. 2016 May 27;6:26840. doi: 10.1038/srep26840 (PMC4882534; doi:10.1038/srep26840)
Supplement: Supplementary Information [file srep26840-s1.pdf]

High-fidelity spherical cholesteric liquid crystal Bragg reflectors generating unclonable patterns for secure authentication :

Supporting Information

Yong Geng[1], JungHyun Noh[1], Irena Drevensek-Olenik[2,3], Romano Rupp[4], Gabriele Lenzini[5] and Jan P. F. Lagerwall[*1]

April 27, 2016

1 Microfluidic production of cholesteric shells

Monodisperse shells and droplets of different cholesteric mixtures were produced using the coaxial glass capillary microfluidic set-up shown in Fig. 1, following the basic design principles of Utada et al.[1] An inner aqueous phase (immiscible with the liquid crystal) that will form the internal droplet of shells is flown from left to right in an inner cylindrical capillary with tapered end. The liquid crystal is flown as the middle phase in the same direction in the interstitial spaces forming between this capillary and a surrounding square capillary, with inner side length equal to the outer diameter of the cylindrical capillary. At the tip of the latter, the liquid crystal meets a second counter-flowing aqueous phase, which will become the outer, continuous, phase. With properly adjusted flow rates, the co-flowing liquid crystal and inner aqueous solution are flow-focused by the external phase, such that an emulsion of the desired shells is picked up in a second tapered cylindrical capillary, extending to the right in the figure. The shell diameter and thickness can be tuned by adjusting flow rates of the different fluids, as well as by varying the orifice size of the tapered capillary. If droplets rather than shells are desired, the flow rate of the inner solution is reduced to zero.

The cylindrical capillaries were tapered by a micropipette puller (P-100, Sutter Instrument) and then cut and fire polished using a Micro Forge (Narishige, MF - 900), allowing us to set the orifice diameter at 40 μm and 150 μm for the injection and collection capillaries, respectively. The outer surface of the injection capillary was treated using octadecyl trichlorosilane (Sigma-Aldrich), whereas the inner surface of the collection capillary was treated with 3-[methoxy (polyethyleneoxy) propyl] trimethoxysilane (90%, 6-9 PE units) (ABCR GmbH), in order to ensure hydrophobic and hydrophilic surface behaviors, respectively.

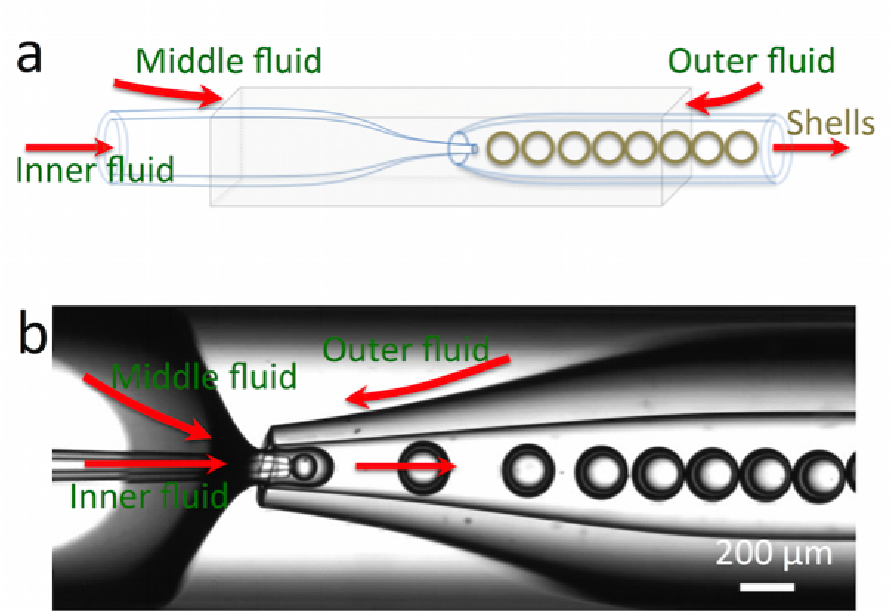

Figure 1: The microfluidic shell production process. Schematic drawing (a) and micrograph (b) of the process for producing cholesteric liquid crystal microshells using the glass capillary microfluidic device. See text for further explanations.

To ensure the radial orientation of the cholesteric helix that is required for the desired omnidirectional Bragg reflection, tangential alignment of the liquid crystal director field at the shell/droplet interfaces is required. To this end, we avoided surfactants as emulsion stabilizers, as these induce radial director alignment, and the shells/droplets were instead stabilized by polyvinylalcohol (PVA, Sigma-Aldrich, M_w: 85,000 - 124,000 g mol^{-1}, 99+% hydrolysed), which does not interfere with the tangential alignment that is promoted by the aqueous isotropic phases in contact with the liquid crystal.[2] The PVA concentration was 15 and 10 wt.-% for the inner and outer fluids, respectively. In order to roughly match the density of these phases to that of the liquid crystal, they contained glycerol (Sigma-Aldrich) and water in a 20/80 volume ratio.

The three fluids were pumped using a microfluidic flow control system (Fluigent MFCS-EZ) to different channels. The applied pressures varied from 20 to 200 μbar. The shell production was conducted at 45°C by mounting the channel in a hot stage, in order to keep the liquid crystal mixture in its isotropic state during the process. A high speed camera (NX4-S3, Integrated Design Tools, USA) coupled to an inverted microscope (TS100, Nikon) allowed optical monitoring of the production process. The emulsion was collected into rectangle capillaries or vials according to the intended purpose.

2 Slow-motion video of the microfluidic shell production process

This is the movie file labeled SI2. Fig. 1 shows a snapshot from the movie.

3 Confirmation of right-handed polarization of communication pattern

To confirm that the communication spots have right-handed polarization (expected since the cholesteric liquid crystal has a right-handed helix) the shells were filled into a capillary and observed in reflection, illuminated by unpolarized light but observed through a $\lambda/4$ plate and analyzer. The latter was rotated such that the $\lambda/4$ plate–analyzer combination acts like a left- and right-handed circular polarizer, respectively. As can be seen in Fig. 2, the reflection spots are almost entirely blocked out when the analyzer is set for transmission of left-handed circular polarized light.

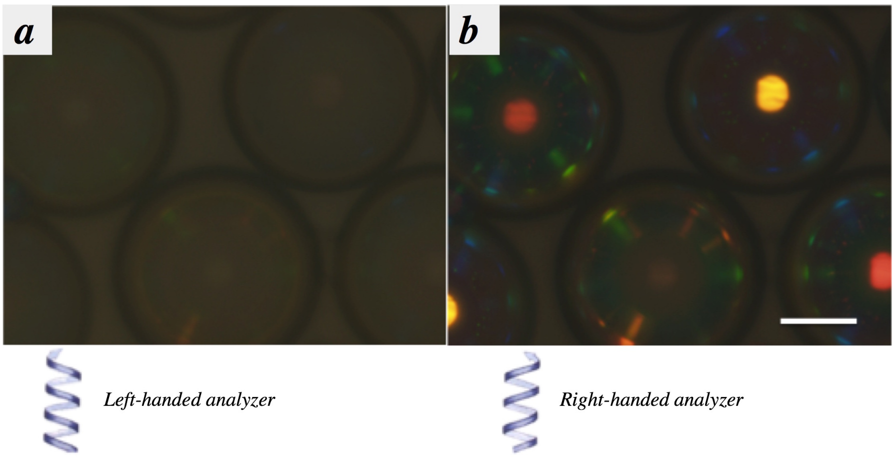

Figure 2: Direct cross communication pattern from cholesteric shells in a capillary as viewed through a left- (a) and right-handed (b) circular polarizer, respectively. The illuminating light is in both cases unpolarized. The scale bar is 50 μm.

4 Interactive simulation of cross communication ray path

The results of the analysis in Section 8 can be explored in an interactive simulation of the cross communication ray path, using any browser, at the URL: https://www.geogebra.org/m/2369963. To tune the simulated system, use the following tools:

- To move the currently shown area, click on a white area and drag.

- To zoom out, scroll upwards; to zoom in, scroll downwards.
- To change the diameter of the left shell (centered around M_1), click on the point labeled 'A' and drag.
- To change the diameter of the right shell, click on the point labeled 'B' and drag.
- To move the center of the left shell vertically, click on the point labeled 'M_1' and drag.
- To move the center of the right shell horizontally, click on the point labeled 'M_2' and drag.
- To move a reflection point on either shell, click on it and drag it.
- To move an observation point (C and D, respectively), click on it and drag it.

5 Video of mechanical deformation and relaxation of polymerized cholesteric shells

This is the movie file labeled SI5. The photos in Fig. 4 in the main article are snapshots from this movie. The shells contain 20% of the reactive mesogen RM-257 and 80% unpolymerizable mesogens (the nematic mixture RO-TN 605 and the chiral dopant CB-15, in a ratio tuned to achieve the desired optical properties).

6 Photonic cross communication between multiple shells and droplets with different cholesteric pitches and of different sizes

As demonstrated and explained in the main text and in Section 8 of this document, photonic cross communication is possible even between shells and droplets of varying size and cholesteric pitches. Fig. 3 shows an area of a sample where multiple shells and droplets (non-polymerized) of different types were brought together in a random arrangement, and the complex multicolored cross communication pattern that develops is a striking demonstration of the rich possibilities of generating unique patterns even with only a few cholesteric microspheres. Three cholesteric liquid crystal mixtures were used, yielding normal reflection very close to infrared (three spheres), in the red-orange (five spheres) and in the yellow range (two spheres). These colors are seen at the center of each sphere, where the illuminating light from above is reflected directly back to the observer.

The peripheral spots reflect the direct cross communication between adjacent spheres (because the sample is in a glass capillary there are no TIR spots) and because a sphere has different types of spheres in its near surrounding, one and the same sphere exhibits multiple colors along its periphery. For instance, the sphere labeled 1 shows cyan-colored

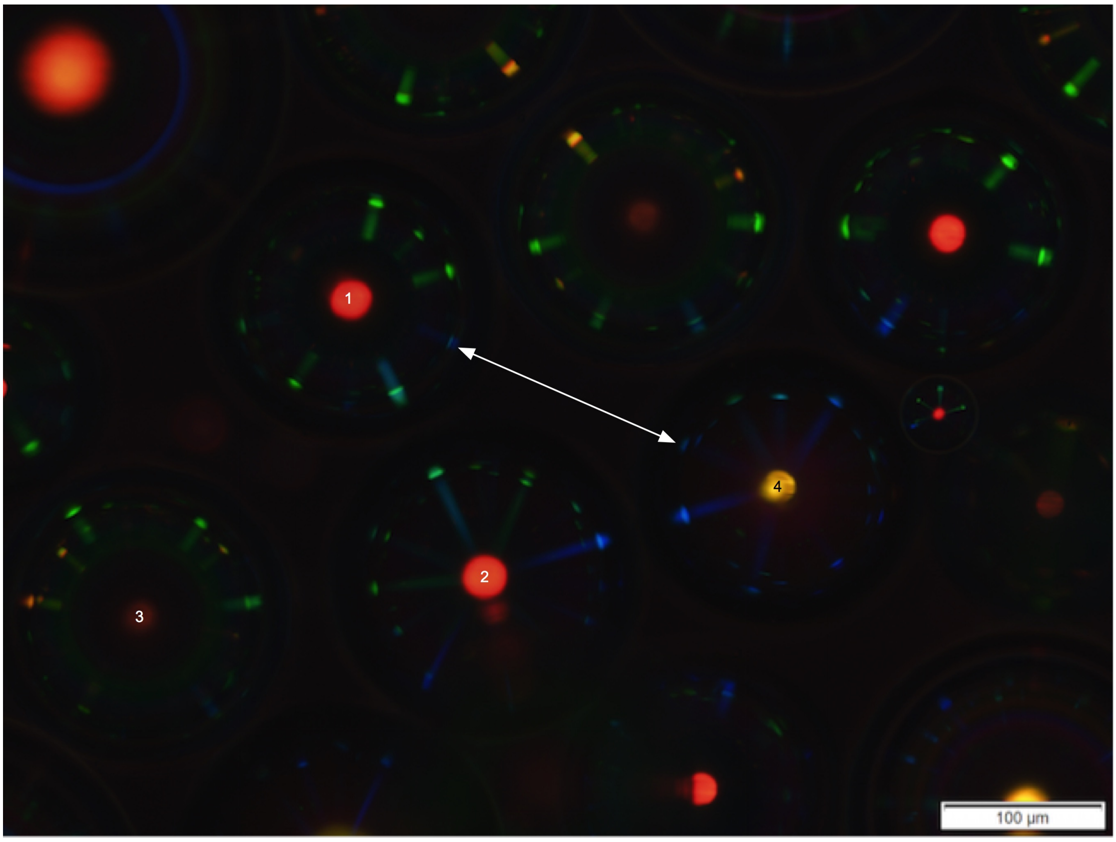

Figure 3: Several cholesteric microspheres with different pitch length and varying sizes arranged randomly, showing photonic cross communication with different wavelengths in different directions.

spots as a result of communication with a sphere from the same normally red-reflecting liquid crystal (labeled 2 in the figure), but when it communicates with normally near-IR-reflecting spheres (e.g. the one labeled 3), the arising spot is green. In one point it communicates with a normally yellow-reflecting sphere (labeled 4), giving rise to a faint blue color, highlighted with a double-headed arrow.

The sphere labeled 3 shows red-orange, yellow-orange and green spots, due to its particular environment, whereas sphere 2 develops cyan, green and blue reflections. Sphere 4, having the shortest cholesteric pitch, shows primarily blue cross communication spots, but also cyan and green can be detected, due to surrounding spheres with longer pitch.

Importantly, the locations of each spot is dictated by the particular relative arrangement of the adjacent spheres. If a sample is prepared with shells of a few different cholesteric mixtures, arranged randomly and not given enough time to organize into a hexagonal colloidal crystal arrangement, the pattern that develops will be characterized by an irregular combination of differently colored spots. The spots are unique in color as well as in location to this particular sample.

The micrograph in Fig. 3 was taken with full sample illumination. By closing the illuminated area around one particular sphere, many spots will darken in this sphere, while the spheres in the surrounding region will still be bright. This dynamic aspect was demonstrated and explained for the case of identical cholesteric droplets in our previous work.[3]

7 Calculation of the effective cone angle in the sample

The reflection cone angle in air, γ_r, is given by the numerical aperture $N.A.$ of the objective, via the formula $N.A. = n \sin \gamma_r$, where n is the refractive index of the medium outside the objective. In our case this is air, with $n \approx 1$. This angle gives the maximum inclination from the vertical direction that a light ray reflected from the sample can have and still be collected by the objective and thus imaged. Because of the change in refractive index at the interface between the glass ($n \approx 1.5$) of the capillary holding the sample and air, the light path is refracted at this point, leading to an even lower effective admittance cone angle γ_r' within the sample. Its value is given by Snell's law: $N.A. = 1 \cdot \sin \gamma = n \cdot \sin \gamma'$. Because the change in (average) refractive index at the interfaces glass–continuous phase and continuous phase–liquid crystal, respectively, is very small, we can neglect further changes to the cone angle.

8 Analysis of the photonic cross communication ray path

8.1 General solution

The geometry of the ray path for reflection at the surfaces of two spheres with radii r_1 and r_2 and centers at M_1 and M_2, respectively, is investigated. The considered plane is defined by the optical axis of the microscope and the distance vector $\vec{d}$ from M_1 to M_2.

The reflection occurs at point A on the first and at B on the second sphere, the pertinent radial vectors from M_1 and M_2, respectively, being $\vec{r}_1$ and $\vec{r}_2$. With the connection vector $\vec{c} = -\vec{r}_1 + \vec{d} + \vec{r}_2$ we may calculate the cosines of the reflection angles:

$$\cos\theta_1 \quad = \vec{r}_1 \cdot \vec{c}/r_1 c \quad = \lambda/\bar{n}P_1 \tag{1}$$

$$\cos\theta_2 \quad = -\vec{r}_2 \cdot \vec{c}/r_2 c \quad = \lambda/\bar{n}P_2 \tag{2}$$

On the right hand side we have included the Bragg condition at wavelength λ, average refractive index $\bar{n}$ and helix pitches P_1 and P_2, respectively. Denoting the positive angle counterclockwise between the radial vector $\vec{r}_1$ and the distance vector $\vec{d}$ by φ_1 and clockwise the positive angle between $\vec{r}_2$ and $-\vec{d}$ by φ_2, respectively, one may write:

$$\cos\theta_1 = \frac{1}{c}\left[-r_1 + d\cos\varphi_1 - r_2\cos(\varphi_1+\varphi_2)\right] \tag{3}$$

$$c = \left[r_1^2 + r_2^2 + d^2 - 2d(r_1\cos\varphi_1 + r_2\cos\varphi_2) + 2r_1 r_2\cos(\varphi_1+\varphi_2)\right]^{1/2}$$

The angle of incidence and the exit angle, respectively, are

$$\alpha \quad = \quad \theta_1 + \varphi_1 - \pi/2$$

$$\beta \quad = \quad \theta_2 + \varphi_2 - \pi/2$$

8.2 Special cases

8.2.1 Touching spheres

For touching spheres of the same radius r the formula eq. (3) simplifies to

$$\cos\theta_1 \quad = \quad \frac{-1 + 2\cos\varphi_1 - \cos(\varphi_1+\varphi_2)}{[6 - 4(\cos\varphi_1 + \cos\varphi_2) + 2\cos(\varphi_1+\varphi_2)]^{1/2}} \tag{4}$$

8.2.2 Touching spheres and equal pitches

If we have, in addition, equal pitches $P_1 = P_2 = P$, it follows from eq. 1 that $\theta_1 = \theta_2 = \theta$, and consequently $\varphi_1 = \varphi_2 = \varphi$. From

$$\cos\theta = \frac{-1 + 2\cos\varphi - \cos(2\varphi)}{[6 - 8\cos\varphi + 2\cos(2\varphi)]^{1/2}} = \frac{(\cos\varphi - \cos^2\varphi)}{[1 - 2\cos\varphi + \cos^2\varphi]^{1/2}} = \frac{(\cos\varphi - \cos^2\varphi)}{1 - \cos\varphi} = \cos\varphi$$

we have $\varphi = \theta$. In this symmetric case the angle of incidence is given by

$$\alpha = \beta = \arccos(\lambda/\bar{n}P) - \arcsin(\lambda/\bar{n}P) \tag{5}$$

From

$$\sin\alpha = 1 - 2(\lambda/\bar{n}P)^2$$

we may deduce in small angle approximation

$$\boxed{\alpha = \beta \;\approx\; 1 - 2(\lambda/\bar{n}P)^2}$$

8.2.3 Differing pitches

In the case of differing pitches we define the reciprocal pitch parameters

$$\begin{aligned} Q_1 &= \frac{\lambda}{\bar{n}} \frac{1}{P_1} \\ Q_2 &= \frac{\lambda}{\bar{n}} \frac{1}{P_2} \end{aligned}$$

and the average and difference parameters

$$\begin{aligned} \bar{Q} &= \frac{1}{2} (Q_1 + Q_2) \\ \Delta Q &= \frac{1}{2} (Q_1 - Q_2) \\ \bar{\varphi} &= \frac{1}{2} (\varphi_1 + \varphi_2) \\ \Delta\varphi &= \frac{1}{2} (\varphi_1 - \varphi_2) \\ \bar{\alpha} &= \frac{1}{2} (\alpha + \beta) \\ \Delta\alpha &= \frac{1}{2} (\alpha - \beta) \end{aligned}$$

and derive from eq.(4)

$$\bar{Q} = \frac{\cos\bar{\varphi}\cos\Delta\varphi - \cos^2\bar{\varphi}}{R} \tag{6}$$

$$\Delta Q = -\frac{\sin\bar{\varphi}\sin\Delta\varphi}{R} \tag{7}$$

$$R = \left[1 - 2\cos\bar{\varphi}\cos\Delta\varphi + \cos^2\bar{\varphi}\right]^{1/2} \tag{8}$$

For small angles of incidence we have $\Delta\varphi \ll \varphi$ and are allowed to approximate:

$$R \approx \left[(1-\cos\bar{\varphi})^2 + \cos\bar{\varphi}\Delta\varphi^2\right]^{1/2} = (1-\cos\bar{\varphi})\left(1 + \frac{1}{2}\cos\bar{\varphi}\Delta\varphi^2\right)$$

It turns out that only eq. (7) has a term linear in $\Delta\varphi$. Therefore all terms proportional to $\Delta\varphi^2$ are neglected and we obtain:

$$\sin\bar{\alpha} \approx 1 - 2\bar{Q}^2 = 1 - \frac{1}{2}(Q_1 + Q_2)^2 \approx \bar{\alpha} \tag{9}$$

$$\sin\Delta\alpha \approx \frac{\Delta Q - Q_2\sqrt{1-Q_1^2} + Q_1\sqrt{1-Q_2^2}}{Q_1 Q_2 + \sqrt{1-Q_1^2}\sqrt{1-Q_2^2}} \approx \Delta\alpha \tag{10}$$

and obtain for the angles of incidence:

$$\alpha = \bar{\alpha} + \Delta\alpha \tag{11}$$

$$\beta = \bar{\alpha} - \Delta\alpha \tag{12}$$

8.3 Discussion

For given pitches P_1 and P_2 the angle of incidence and the exit angle α and β, respectively, can be calculated from eq.s (11) and (12), respectively, for a given wavelength λ. The setting of the contrast diaphragm of the microscope by which the reflections are observed determines the aperture angle of the incident rays and thus the wavelength range observed. We assume here that the illumination aperture is smaller than the observation aperture, as usually is the case. In order to restrict the reflected wavelength range one has to close the contrast diaphragm of the microscope as far as possible. Otherwise one sees a broader spectral range.

9 Spectrophotometric determination of selective reflection and cross communication wavelengths

To obtain estimates for the selective reflection and cross communication wavelengths the reflection patterns were analyzed spectrophotometrically with an Avantes AvaSpec-2048 spectrophotometer connected to the microscope via fiber-optics. The fundamental selective reflection (incidence along the helix axis) gives rise to strong peaks which can be fitted with a Lorentz peak function in the visible range (Fig. 4a and b). The IR-reflecting mixture has a fundamental reflection that is outside the measurement range of the spectrophotometer, and therefore its fundamental reflection wavelength $\lambda^f_{IR} = \bar{n}P_{IR}$ was established by estimating the direct reflection peaks (image in Fig. 5a in the main paper, spectrum in Fig. 4c in this SI file) and calculating λ^f_{IR} from the Bragg relation for direct communication, $\lambda^c_{IRIR} = \lambda^f_{IR} \cos 45°$, yielding a value of about 0.96 μm.

While the symmetric communication peaks λ^c_{RR} and λ^c_{GG} can be calculated from the Bragg relation for direct communication using the fundamental reflection wavelengths λ^f_R and λ^f_G, we must use the raw spectrophotometric data to establish the wavelengths of the asymmetric communication spots in Fig.5d-f in the main paper. Because all communication spots are very small and weak, a rather special spectrophotometer, with very high sensitivity and capable of localized analysis, is required to resolve sharp spectra for communication spots.[4] Unfortunately we did not have access to such a spectrophotometer in this study, and the spectra obtained are therefore averages over the whole image, with very weak peaks and much background noise. Nevertheless, as we know the color of each peak from visual inspection we can identify the peak in the spectrum, although the data is too noisy to carry out regular curve fitting. Instead we have adjusted a Lorentz peak function manually (using the software ProFit, QuantumSoft) to highlight the peak in each diagram in Fig. 4c-f below. The adjustment procedure also allows us to extract estimates for the cross communication wavelengths with sufficient accuracy for the discussion around Fig. 5 in the main paper. Note that the cross communication takes place over a certain range of angles, as explained in the main paper, and the angles and wavelengths indicated are thus only examples of communication pathways. There are many alternative combinations with variations around the estimated wavelengths and corresponding angles.

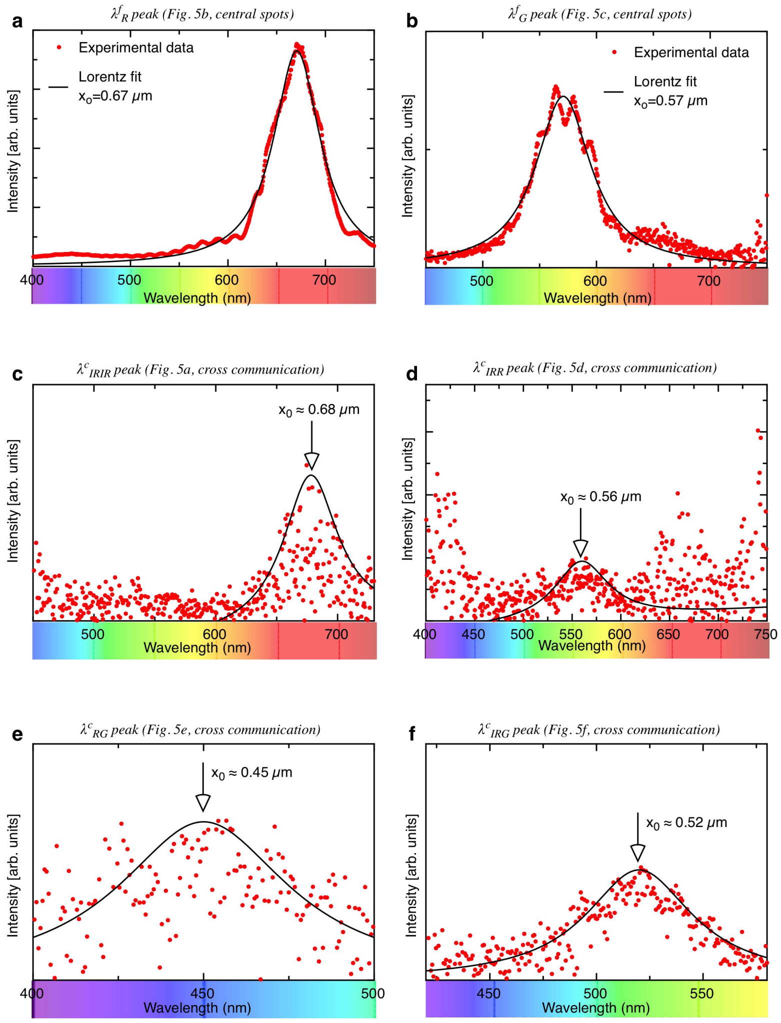

Figure 4: Spectra showing the fundamental reflection (a-b; peak wavelengths obtained by fitting) and the photonic cross communication between shells (c-f). Note that the curves in c-f are not obtained by fitting, but manually adjusted to provide guides for the eye, highlighting the peak of interest in the rather noisy data.

References

[1] A. Utada, E. Lorenceau, D. R. Link, P. D. Kaplan, H. A. Stone, and D. A. Weitz, "Monodisperse double emulsions generated from a microcapillary device," *Science*, vol. 308, no. 5721, pp. 537–541, 2005.

[2] J. Noh, K. Reguengo De Sousa, and J. P. F. Lagerwall, "Influence of interface stabilisers and surrounding aqueous phases on nematic liquid crystal shells," *Soft Matter*, vol. 12, no. 2, pp. 367 – 372, 2016.

[3] J. Noh, H.-L. Liang, I. Drevensek-Olenik, and J. P. F. Lagerwall, "Tuneable multicoloured patterns from photonic cross communication between cholesteric liquid crystal droplets," *J. Mater. Chem. C*, vol. 2, no. 5, pp. 806–810, 2014.

[4] J. Noh, I. Drevensek-Olenik, J. Yamamoto, and J. P. F. Lagerwall, "Dynamic and complex optical patterns from colloids of cholesteric liquid crystal droplets," *Proc. SPIE*, vol. 9384, Emerging Liquid Crystal Technologies X, p. 93840T, 2015.
